# Supplementary material for: FastqPuri: high-performance preprocessing of RNA-seq data
Source: BMC Bioinformatics. 2019 May 3;20:226. doi: 10.1186/s12859-019-2799-0 (PMC6500068; doi:10.1186/s12859-019-2799-0)
Supplement: Supplementary file 2 — Archive of FastqPuri. Archive containing all files needed to install and run FastqPuri v1.0.6. Date stamp March 22, 2019. (GZ 47,819 kb) [file 12859_2019_2799_MOESM2_ESM.gz › FastqPuri-1.0.6/html/pages.html]

My Project: Related Pages


My Project


Related Pages

Here is a list of all related documentation pages:

|  |  |
| --- | --- |
| Installing Pandoc |  |
| FastqPuri, an fq quality control and filter tool |  |
| makeBloom user manual |  |
| makeTree user manual |  |
| QReport user manual |  |
| SReport user manual |  |
| trimFilter user manual |  |
| trimFilterDS user manual |  |


---

Generated by  

 1.8.14
